# HBV genotype distribution and S gene mutations in HIV-HBV co-infected patients: insights from North India

**DOI:** 10.3389/fcimb.2025.1731472

**Published:** 2026-02-09

**Authors:** Hiba Sami, Mohd Asaad, Syed Haider Mehdi Husaini, Parvez Anwar Khan, Adil Raza, Nazish Fatima, Haris M. Khan

**Affiliations:** 1 Department of Microbiology, Jawaharlal Nehru Medical College, Aligarh Muslim University, Aligarh, India; 2 Department of Medicine, Jawaharlal Nehru Medical College, Aligarh Muslim University, Aligarh, India

**Keywords:** ayw2, HBV genotyping, HBV-HIV coinfection, S gene mutations, serotypes

## Abstract

**Purpose:**

Effective HBV therapy and immunisation require knowledge of HBV mutation rate and genetic variability. This study aimed to characterize HBV genotypes, serotypes, and surface (S) gene mutations among HBV–HIV co-infected and HBV mono-infected patients in North India.

**Methods:**

A total of 100 HBV–HIV co-infected and 50 HBV mono-infected patients were enrolled. HBV DNA was extracted from serum using the QIAamp DNA Blood Mini Kit (QIAGEN). HBV viral load was quantified by real-time PCR. The HBV surface gene was amplified using conventional and nested PCR, followed by Sanger sequencing for genotyping, serotyping, and mutational analysis. Genotypes were additionally confirmed using type-specific multiplex PCR.

**Results:**

The mean age of HIV-HBV co-infected and HBV mono-infected patients was 36.6 and 34.9 years, respectively. HBeAg positivity was observed in 17% of co-infected and 10% of HBV mono-infected cases. HBV surface gene was successfully amplified in 19 (19%) HIV-HBV co-infected and 20 (40%) HBV mono-infected samples. Genotype D predominated (35/39, 89.7%), with subgenotypes D1 and D3 detected at comparable frequencies, while genotype A was identified in 4 (10.3%) samples, all belonging to subgenotype A1. Serotype analysis showed exclusive association of genotype D with ayw2 and genotype A with adw2. Mutations within the HBsAg ‘a’ determinant region were identified in 36.8% of HBV–HIV co-infected patients, including nonsynonymous substitutions, whereas no such mutations were observed in HBV mono-infected individuals. Genotype D sequences clustered with subgenotypes D2, D3, and D9 (from West Bengal), as well as D3 (from Russia, Madhya Pradesh, Delhi and Brazil).

**Conclusion:**

This study confirms the predominance of HBV genotype D with serotype ayw2 in North India and demonstrates a higher frequency of HBsAg immune escape–associated mutations in HBV–HIV co-infected patients. These findings underscore the importance of ongoing molecular surveillance to inform diagnostic strategies, vaccination effectiveness, and clinical management of HBV, particularly in HIV-affected populations.

## 1 Introduction

According to the latest WHO Hepatitis B fact sheet, an estimated 254 million people worldwide were living with chronic hepatitis B virus (HBV) infection in 2022, highlighting the significant worldwide impact of this disease. India alone harbours a significant portion of this burden, with approximately 40 million people living with chronic HBV infection (World Health Organization, 2025). In India, HBV vaccination was introduced into the Universal Immunisation Programme in 2011, and national coverage with the three-dose HBV vaccine has steadily increased, exceeding 85–90% in recent years, although regional and sub-national disparities persist, particularly in northern India (World Health Organization, 2019). Concurrently, India has a substantial HIV burden, with approximately 23.49 lakh individuals living with HIV/AIDS (HIV Facts & Figures, 2019). In northern India, HBV prevalence is particularly high, and HBV-HIV co-infection rates have been reported to reach 7.1% (HIV). Understanding the genetic diversity of HBV in both HBV mono-infected and HIV-HBV co-infected individuals is essential for enhancing clinical management, improving treatment outcomes, and informing public health strategies.

Classically, there are four primary serotypes based on the major antigenic determinants of the surface antigen (HBsAg): adw, adr, ayw, and ayr. However, with more detailed serological studies, up to nine serological variants have been described (e.g., adw2, adw4, adrq+, adrq–, ayw1, ayw2, ayw3, ayw4, and ayr) based on finer differences in HBsAg antigenicity (Datta, 2008; Mlewa et al., 2025). Because of its distinctive life cycle that involves an error-prone reverse transcriptase for replication, the virus continually evolves, leading to significant genetic diversity in genotypes, sub-genotypes, and mutations (Zhang et al., 2016). HBV genomes have significant sequence variation, comprising at least 10 genotypes (A to J) and over 40 known genetic subtypes (Kramvis et al., 2005). In India, genotype D is the most widespread, followed by genotype A, with genotype C being the least common in the north region (Sami et al., 2013; Chen et al., 2023).

In immunocompetent individuals, most acute HBV infections resolve spontaneously; however, progression to chronic infection is strongly age-dependent, occurring in approximately 90% of infants infected within their first year, ~30% of children infected between 1 and 5 years of age, and only 5–10% of individuals infected after 5 years of age (Pattyn et al., 2021). In contrast, people living with HIV (PLWH) have a lower clearance rate, leading to persistent HBV replication and worse outcomes due to immunosuppression. Also, there is increasing evidence that certain mutations in the HBV viral genome are higher in HIV-HBV co-infected individuals and favour the rapid progression of liver disease in these individuals (Singh et al., 2017).

Despite the high prevalence of HBV infection in North India, extensive data on regional HBV molecular diversity, specifically regarding surface gene variants, serotypes, and genotype distribution in HBV co-infected with HIV, is scarce. Due to the significant HIV prevalence in this area and the influence of HIV-related immunosuppression on HBV evolution, region-specific molecular characterisation is crucial for enhancing diagnostic accuracy, directing antiviral treatment, and guiding targeted public health measures. This study aims to characterize the genetic variability of the HBV surface gene, genotypes, sub genotypes, serotypes among HBV mono-infected and HBV-HIV co-infected patients in North India.

## 2 Methodology

This was a hospital-based cross-sectional observational study conducted at a tertiary care centre in North India.

### 2.1 Study setting

This study was conducted at a tertiary care teaching hospital in North India that serves as a referral centre for patients attending the antiretroviral therapy (ART) clinic and Integrated Counselling and Testing Centre (ICTC). Patient recruitment and sample collection were carried from June 2021 to December 2023.

### 2.2 Study design

This was a hospital-based cross-sectional observational study designed to characterize HBV genotypes, serotypes, and surface gene mutations among HBV–HIV co-infected and HBV mono-infected patients.

### 2.3 Study population

**Figure 1:**
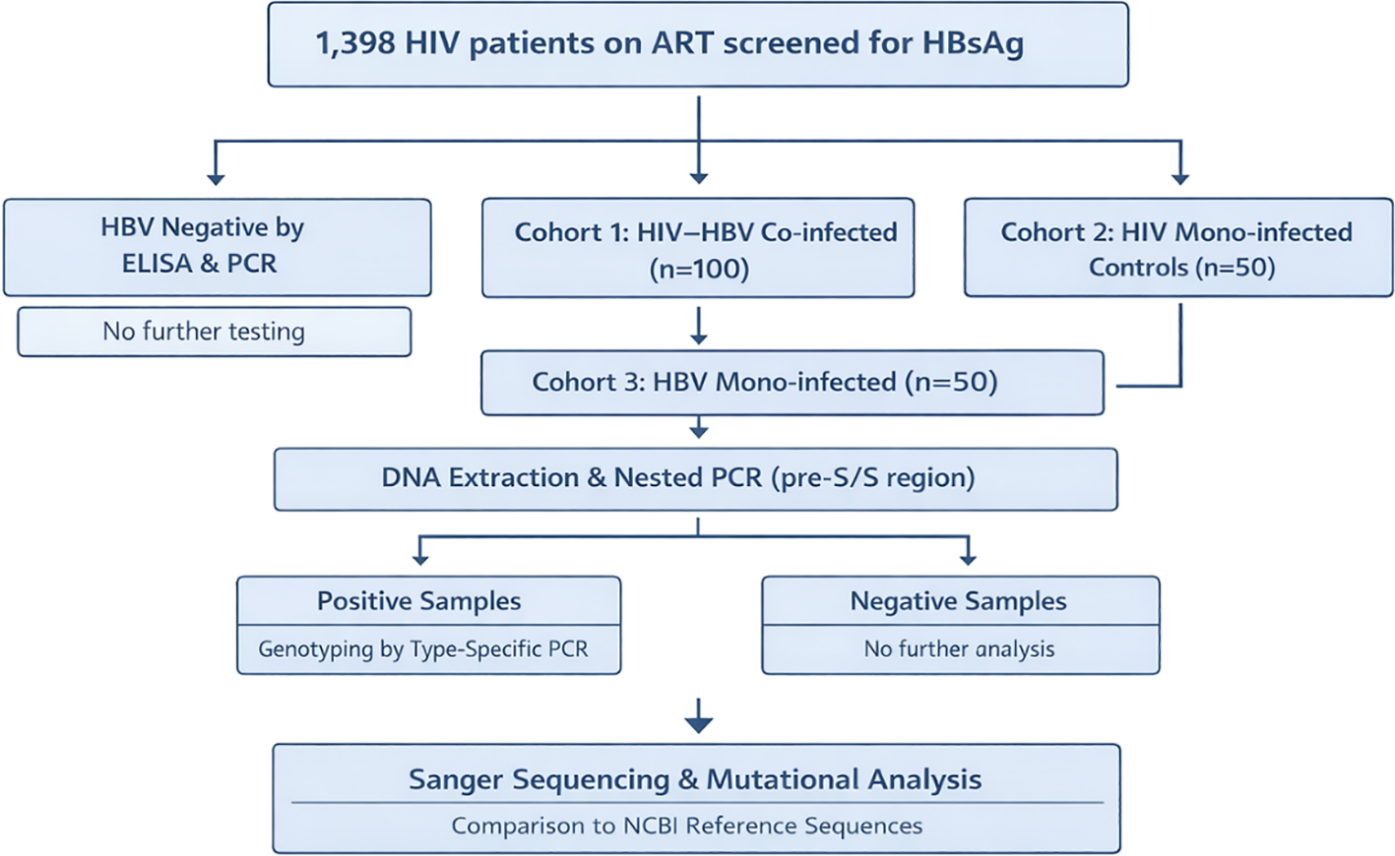
Flow chart showing selection and processing of HIV/HBV co-infected and HBV mono-infected patient samples for HBV Surface region mutational analysis.

The study population comprised: 100 HIV–HBV co-infected patients, and 50 HBV mono-infected patients (**Figure 1**), who were recruited after obtaining written informed consent. HIV–HBV co-infection was defined by documented HIV infection with HBsAg positivity and/or detectable HBV DNA, while HBV mono-infection was defined by HBsAg positivity in the absence of HIV infection. 1,398 HIV-infected patients with liver disease symptoms were screened for HBV. HBV was detected in 100 co-infected patients (Prevalence 7.2%).

### 2.4 Sample size calculation

As the prevalence of HIV-HBV coinfection in HIV patients was found 6.02% from previous North Indian study, sample size is calculated by,

Sample size = Z_1−a/22_p(1-p)

d_2_

Here

Z_1−a/2_ = Is standard normal variate (at 5% type 1 error (P<0.05) it is 1.96

p = Expected proportion in population based on previous studies or pilot studies

d = Absolute error or precision

Considering precision/absolute error of 5% and at type 1 error of 5%,

Sample size = (1.96)_2_0.06(1-0.06) = 86.6

0.05_2_

### 2.5 Sampling technique

A consecutive (non-probability) sampling method was employed, whereby all eligible patients meeting the inclusion criteria and presenting during the study period were enrolled until the desired sample size was achieved. This approach was chosen due to the hospital-based nature of the study and the relatively low prevalence of HIV–HBV co-infection.

### 2.6 Inclusion and exclusion criteria

Positive HBsAg, HBeAg, or HBV DNA results confirmed HBV status. Patients were excluded if they had documented evidence or clinical history of liver disease due to causes other than HBV infection, including other viral hepatitis (HAV, HCV, or HEV), autoimmune hepatitis, alcohol-related liver disease, or drug-induced liver injury. Patients with known metabolic or inherited liver disorders or those not providing informed consent were also excluded.

### 2.7 Ethical approval

The study was approved by Institutional Ethics Committee, with reference number IEC/JNMC/224 dated 14.12.2020.

### 2.8 Sample collection and storage

Venous blood samples were collected under aseptic conditions. Serum was separated and stored at –20°C until further analysis.

### 2.9 Serological testing

HBV serological markers (HBsAg, anti-HBc-IgM and anti-HEV IgM) were tested using commercially available ELISA kits (QUALISA, India and DIA.PRO, Italy). HIV diagnosis followed the national triple-test strategy at the ICTC: initial screening with Comb-Aids (Arkray Healthcare), followed by Tri-Line (Meriscreen) and Tri-Dot (Tredro) assays.

### 2.10 Assessment of liver disease severity

Liver fibrosis was assessed using two non-invasive indices: AST to Platelet Ratio Index (APRI) and Fibrosis-4 (FIB-4) score, calculated as follows: APRI Score: (AST [IU/L]/upper normal limit of AST [IU/L])/platelets [10^3^/mm^3^] (Lin et al., 2011) FIB-4 Score: (Age [years] × AST [IU/L])/(platelets [10³/mm³] × √ALT [IU/L]) (Sterling et al., 2006).

### 2.11 DNA isolation and HBV viral load

DNA was extracted from 200 ml of serum using the QIAamp DNA Blood MiniKit (QIAGEN, Hilden, Germany). HBV viral load detection was done using a commercially available kit AltoStar HBV PCR Kit 1.5 (Altona Diagnostics, Hamburg, Germany). Viral load was calculated based on the Ct value obtained using the Bio-Rad CFX96 Touch Real-Time PCR Detection System, following the manufacturer’s protocol. The viral load in IU/mL was determined using the formula:

Viral Load (sample) [IU/mL] = (Elution Volume [µL] × Viral Load in Eluate [IU/mL])/Sample Input Volume [mL].

### 2.12 Amplification of surface gene region

The HBV surface gene region was amplified using nested polymerase chain reaction (PCR). In the first round, 2 μL of extracted DNA was added to a 20 μL PCR reaction mixture containing forward primer HS1F (5′-YCCTGCTGGTGGCTCCAGTTC-3′) and reverse primer HSR (5′-AAGCCANACARTGGGGGAAAGC-3′). Subsequently, 2 μL of the first-round PCR product was used as the template in a second-round PCR with primers HSF2 (5′-GTCTAGACTCGTGGTGGACTTCTCTC-3′) and the same reverse primer HSR.

PCR cycling conditions were 96°C for 2 min for initial denaturation, 25 cycles of 96°C for 15 sec., 58°C for 45 s, and 72°C for 45 s, and a final extension at 72°C for 7 min using a Bio-rad (C1000 Touch) 96 well thermal Cycler. The PCR reaction mixture concentration and conditions were similar for both the first and second-round PCR. PCR amplicons were electrophoresed on 1.5% agarose gel with ethidium bromide staining and visualized under UV. The expected amplicon sizes were approximately 680 bp for the outer region and 412 bp for the nested (inner) region, corresponding to the pre-S and S gene regions of HBV.

### 2.13 Genotyping using type specific PCR

HBV-positive samples were genotyped using multiplex PCR with primers published by Kirschberg et al. (Kirschberg et al., 2004), following the protocol described by Hiba et al. (Sami et al., 2013). The expected amplicon sizes were: Genotype A: 370 bp, B: 190 bp, C: 701 bp, D: 147 bp, E: 787 bp, and F: 481 bp. The determination of HBV strain genotypes predominantly relied on the confirmation through analysis of the surface gene sequence within the HBV genome.

### 2.14 Sequencing, mutation and genotype analysis

Purified PCR products of the amplified large surface gene (pre-S1, pre-S2, and part of the small S gene) were sequenced by Sanger sequencing (Eurofins Genomics Pvt. Ltd.). The sequences were assembled, annotated, and aligned using UGENE v2.0. Mutational analysis was performed by calculating synonymous (ds) and non-synonymous (dn) substitution rates, along with the proportions of each (ps, pn), following the method of Ota and Nei (1994). Variant calling was conducted using the “Find Variation/SNP” tool in Geneious. Sequence alignment was done using ClustalW. HBV Serotyping was done using gene sequencing.

HBV genotyping was performed using three approaches: comparison with reference sequences from GenBank, the NCBI HBV genotyping tool, and jpHMM (Schultz et al., 2012). Final confirmation was done via phylogenetic tree construction.

### 2.15 Phylogenetic analysis

Phylogenetic analysis was performed on 39 HBV S gene sequences (19 HIV-HBV co-infected, 20 HBV mono-infected) using ClustalW for alignment and the Neighbour-Joining method in UGENE v2.0 under the Maximum Composite Likelihood model with 1,000 bootstrap replicates. Reference sequences for genotypes A–J were retrieved from GenBank, including 18 for genotype D (covering subgenotypes D1–D10). The tree was cross-validated with NCBI data to identify circulating genotypes based on genetic clustering (Tamura et al., 2011).

### 2.16 Recombination analysis

Recombination study that was conducted on the sequences using the jumping profile Hidden Markov Model (JPHMM) with the help of an online tool (http://jphmm.gobics.de/), following the methodology outlined by Schultz et al (Schultz et al., 2012).

### 2.17 Accession numbers

All 33 HBV surface gene sequences generated in this study were submitted to the NCBI GenBank database and assigned accession numbers PP504744-PP504776. The sequence data have been archived and are freely available on GenBank.

### 2.18 Statistical analysis

Statistical analysis was performed using IBM SPSS Statistics version 19. Normally distributed data were presented as means ± standard deviation (SD) and compared using independent-samples t-tests or one-way ANOVA. Non-normally distributed data were expressed as medians with interquartile ranges (IQR) and analysed using the Mann–Whitney U test. Associations between categorical variables—including HBsAg status and HIV transmission routes, HBeAg positivity, CD4 immunosuppression status, and liver injury markers (ALT, AST, APRI, and FIB-4 indices)—were assessed using the Chi-square test. A p-value of <0.05 was considered statistically significant.

## 3 Results

**Figure 2:**
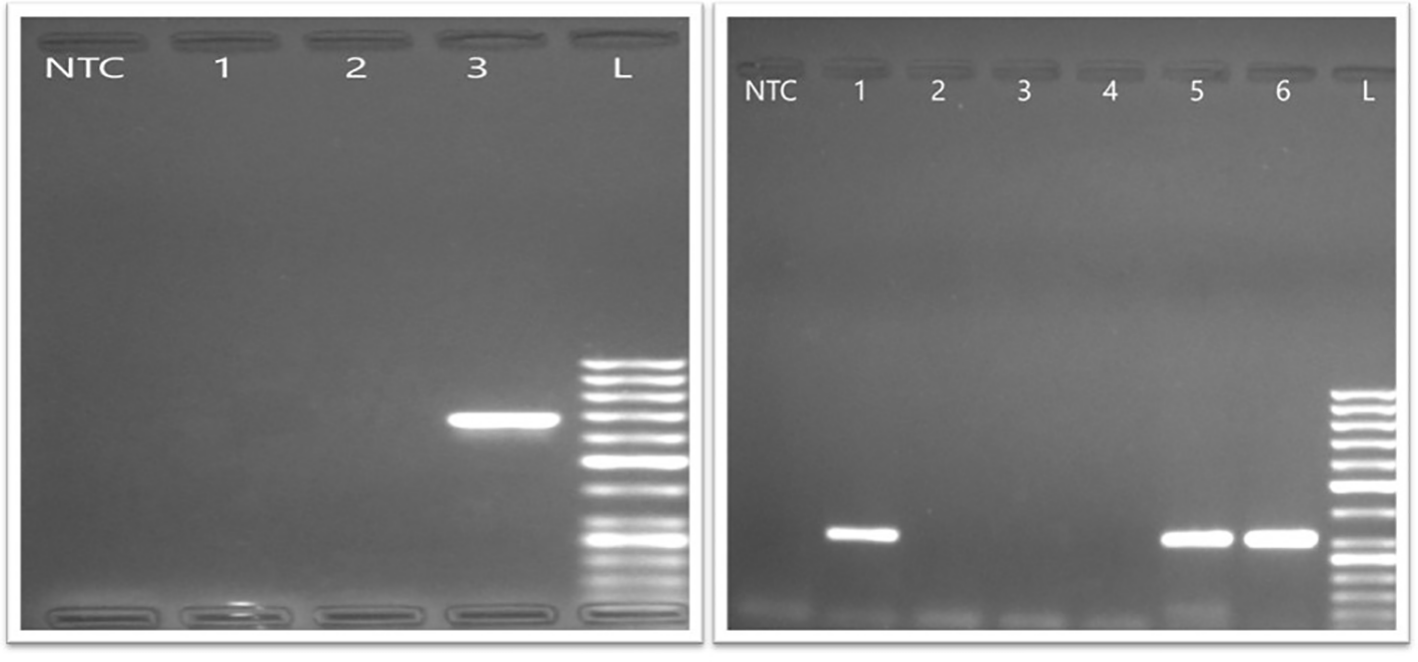
Agarose gel electrophoresis results of the S gene. Lane NTC represents the non-template control, while lanes 1, 2, and 3 show PCR products. Lane L denotes the DNA ladder for both the left (amplification of outer region of S gene- 685 bp) and right (amplification of inner region of S gene- 480 bp) images. However, lanes 1 and 2 in the left image, as well as lanes 2, 3, and 4 in the right image, did not exhibit successful amplification.

Among the 100 HBV-HIV co-infected patients the mean age was 36.59 years (Female: Male : Transgender = 29:70:1), while HBV mono-infected patients had a mean age of 34.92 years (F:M = 19:31) as shown in **Table 1**. Conventional PCR successfully amplified the HBV surface (S) gene region (nt 107–718) in 19% of co-infected and 40% of HBV mono-infected samples (**Figure 2**), which were further confirmed by real-time PCR and subjected to Sanger sequencing. Patients in whom HBV surface gene amplification was successful had significantly higher HBV DNA levels and higher HBeAg positivity compared with those in whom amplification failed, both among HIV–HBV co-infected and HBV mono-infected individuals as shown in **Supplementary Table 2**. In contrast, age, liver enzyme levels (ALT, AST, alkaline phosphatase), APRI, FIB-4 scores, and ART duration did not differ significantly between sequenced and non-sequenced patients.

**Table 1:** Demographic and bio-chemical characterization of HBV patients with mono-infection (n=50) and HIV co-infection (n=100).

Characterstics	HIV-HBV Co-infected	HBV mono-infected	P value
Age (Mean)	36.59 ± 14.7	34.92 ± 14.72	.034
Gender	Female: Male : Transgender = 29:70:1	F:M = 19:31	
S Gene Amplified	19 (19%)	20 (40%)	
ALT Mean (95%CI)	62.13 IU/L	123.16 IU/L	.80
AST Mean (95%CI)	55.97 IU/L	94.36 IU/L	.084
Alk. P Mean (95%CI)	167.54 IU/L	176.09 IU/L	.766
APRI Score			.004
<1.0	92 (92%)	35 (70%)	
>1.0	8 (8%)	15 (30%)	
FIB-4			.163
<2.0	74 (74%)	19 (38%)	
>2.0	26 (26%)	31 (62%)	
HBeAg positive			
Positive	17 (17%)	5 (10%)	.465
Negative	83 (83%)	45 (90%)	
HBV Viral load			.550
Target not detected	46 (46%)	8 (16%)	
<20000 IU/ML	24 (24%)	16 (32%)	
>20000 IU/ML	30 (30%)	26 (52%)	
Mean HBV Viral Load (IU/mL)	2.84 × 10^8^	3.20 × 10^8^	

### 3.1 Virological characteristics

The overall HBeAg positivity rate was 17% in HBV-HIV coinfected patients and 10% in HBV mono-infected and there was no statistically significant difference in HBeAg positivity among the two groups (**Table 1**). The overall HBV viral load among 100 HBV–HIV co-infected patients had an IQR of 2.0–9.2 log IU/mL, and the mean viral load was 2.84 × 10^8^ IU/mL. Viral load was higher in HBeAg-positive patients (IQR: 5.4–8.3 log IU/mL) than in HBeAg-negative patients (IQR: 3.0–4.7 log IU/mL) and this difference is significant statistically.

In comparison, HBV mono-infected patients had an IQR of 3.4–7.3 log IU/mL and a mean viral load of 3.20 × 10^8^ IU/mL. Overall, the IQR was lower in HBV–HIV co-infected patients than in HBV mono-infected patients. The mean CD4 T-cell count in the HIV mono-infected patients was 433 cells/μL, while in the HIV-HBV co-infected group, it was 356 cells/μL.

### 3.2 S-Region mutations and prevalent genotypes and serotypes

**Figure 3:**
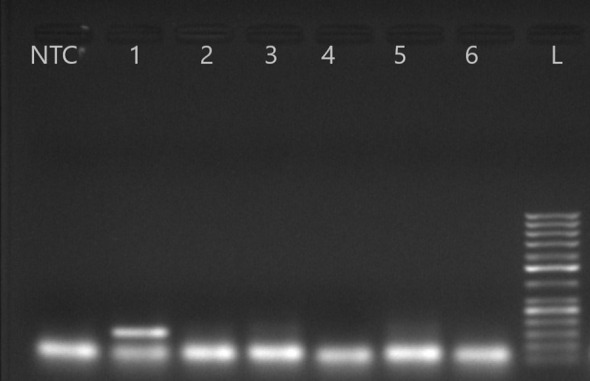
Agarose gel electrophoresis results of the HBV genotype D using primers of Kirschberg et al., 2004 (Singh et al., 2017). PCR product of 147 bp. Lane L:1000 bp DNA ladder; lane NTC: Negative Template Control; lanes 1-6: samples; lanes 2: genotype D.

Multiplex PCR with type-specific primers identified genotype D in 12 (63.1%)HIV-HBV co-infected and 10 (50%) HBV mono-infected samples (**Figure 3**). Sanger sequencing confirmed that genotype D was the most prevalent overall (89.7%), found in 16 (84.2%) HIV-HBV co-infected and 19 (95%) HBV mono-infected patients. Genotype A was detected in 3 (15.7%) HIV-HBV co-infected and 1 (5%) HBV mono-infected sample.

**Figure 4:**
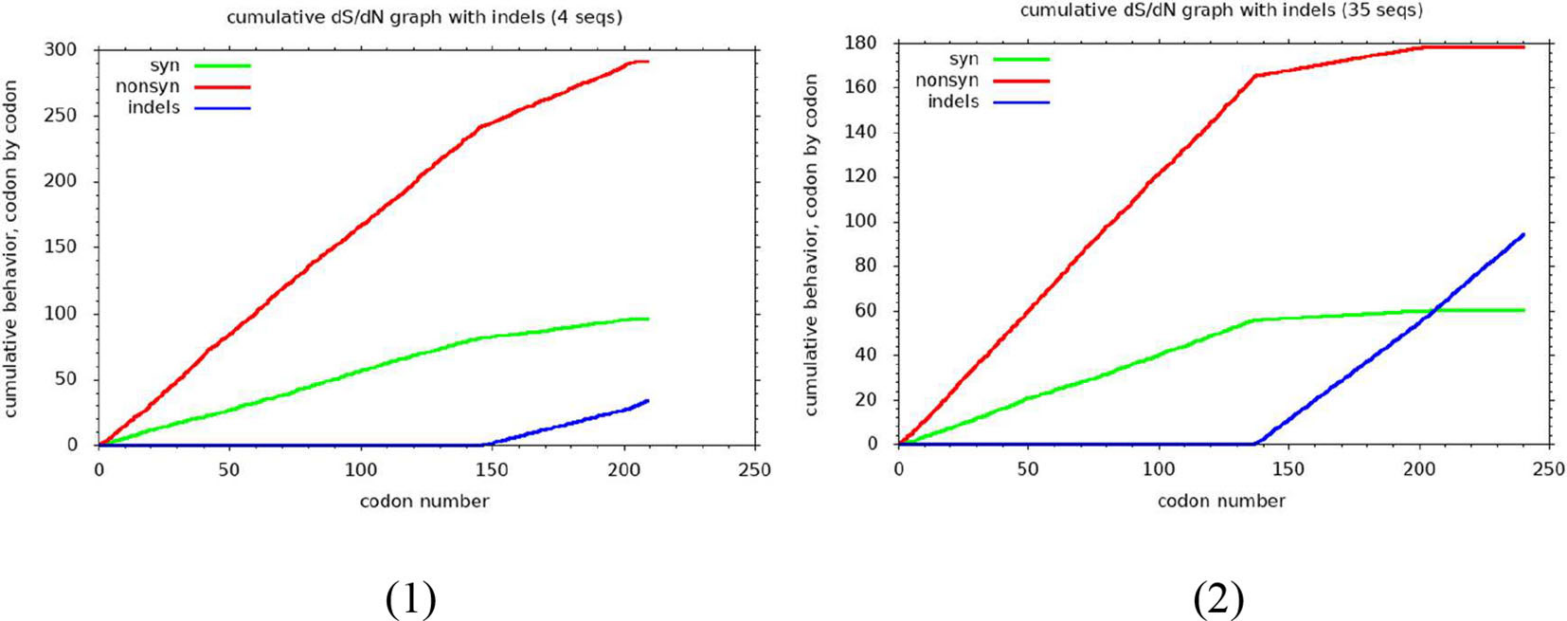
The graph showing the count of synonymous and non-synonymous base substitutions, following the Nei and Gojobori method, for all pairwise sequence comparisons within the alignments of Genotype A (1) and Genotype D (2). The analysis involves tallying the number of synonymous and non-synonymous codon changes, including potential changes when compared with their respective reference sequences. Codons with ambiguity or insertions are excluded from the comparison. The overall sequence distances are computed, and a detailed summary is provided on a codon-by-codon basis. Nei and Gojobori 1986, Ota and Nei 1994, Ganeshan et. al., J Virol 71: 663-677 (1997).

On serotyping using gene sequencing, all samples with genotype D exhibited the presence of serotype ayw2 while genotype A samples belonged to serotype adw2. Within the ‘a’ determinant region of the S region between amino acid residues 99 and 169 (Ruiz-Tachiquín et al., 2007), we identified nucleotide substitution mutations in 7 (36.8%) HIV-HBV co-infected samples. However, no such mutations were found in HBV mono-infected samples. Among these, two mutations led to changes in the amino acid sequences (nonsynonymous), while the remaining five mutations did not impact the amino acid sequences (synonymous) (**Supplementary Table 1**; **Figure 4**).

**Supplementary Figure 1** shows the multiple alignment of the inner region (480bp) (nt 390 to nt 462) of the surface gene of HBV in study samples and genotype specific wild-type references using UGENE software.

Seven mutations were classified as pre-S deletion mutations: All genotype D sequences (preS) had a deletion of nucleotide A from position nt-121; one sample had a G-nucleotide deletion from position 170 (S aa57) and one had an A-nucleotide deletion from position 229 (S aa76) and a-nucleotide deletion from position 455 (S aa151). A-nucleotide deletion from position 459 (S aa 153) was found in three genotype D samples; G-nucleotide deletion from position 465 (S aa155) was found in one sample; and A-nucleotide deletion from position 576 (S aa192) was found in one sample. Both genotype D samples showed an in-frame shift of 3 nucleotides (1 aa) from 281-282. Only some substitution changes were found in eight samples with four patterns (CCATT, CCAGT, CCART, and CCAAG). The CCAAT box had no deletions.

**Figure 5:**
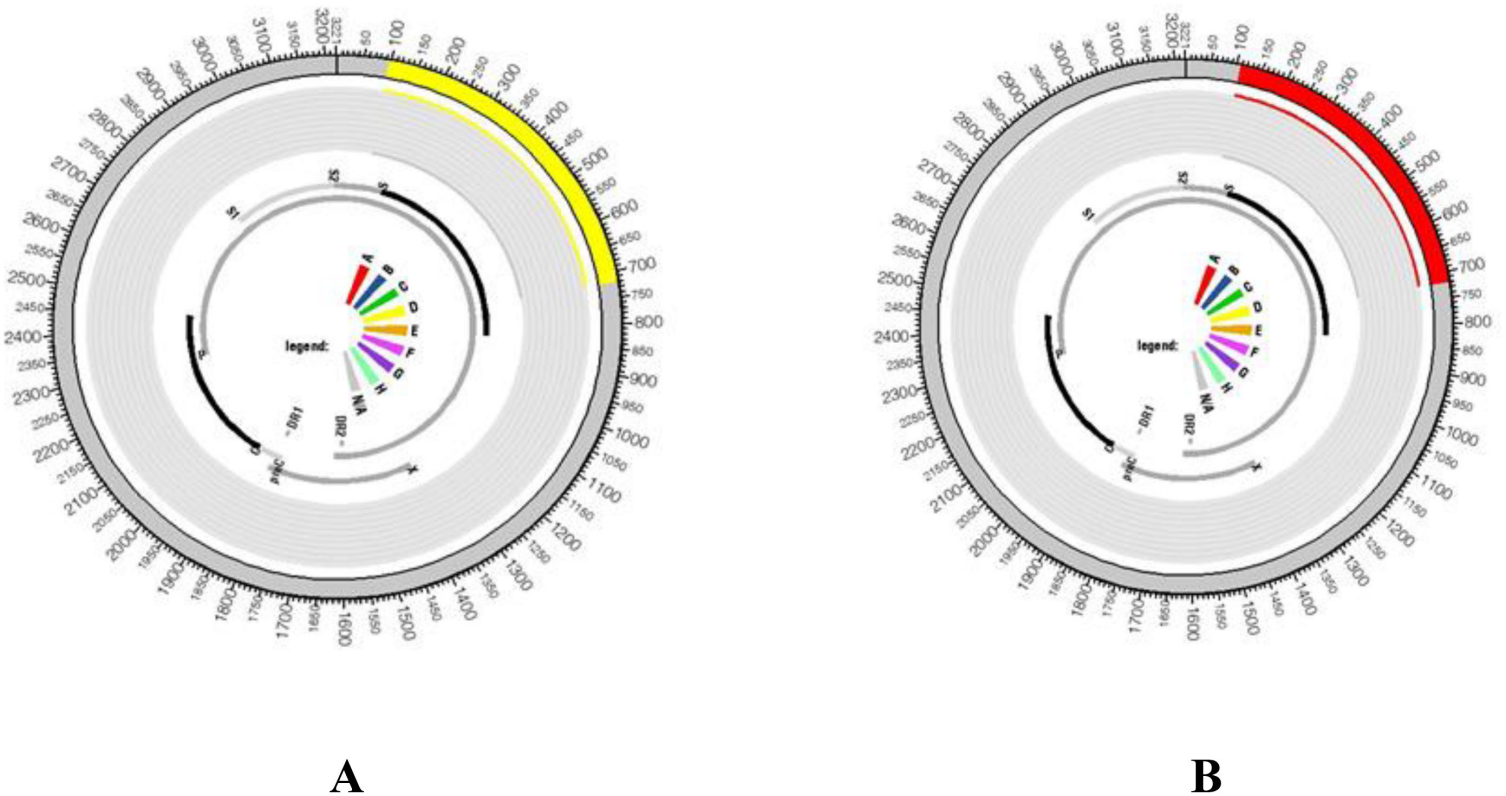
Analysis of genome recombinations was conducted for two HBV isolates, specifically **(A)** HBV/D and **(B)** HBV/A. Schematic diagrams generated from jpHMM (an online tool) results illustrate the circular HBV genome, delineating regions associated with genotype A and genotype D The outer ring of the diagram visually depicts the predicted genotype recombination. In this representation, regions corresponding to genotype D are highlighted in yellow, while those associated with genotype A are marked in a distinct red colour on the HBV genome.

We used jpHMM, a graphical display sequence analysis programme, to examine the sequences and validate the intergenotypic recombination. As shown in **Figure 5**, Our findings indicate that two HBV genotypes—D and A—are circulating in the study region.

### 3.3 Phylogenetic analysis:

**Figure 6:**
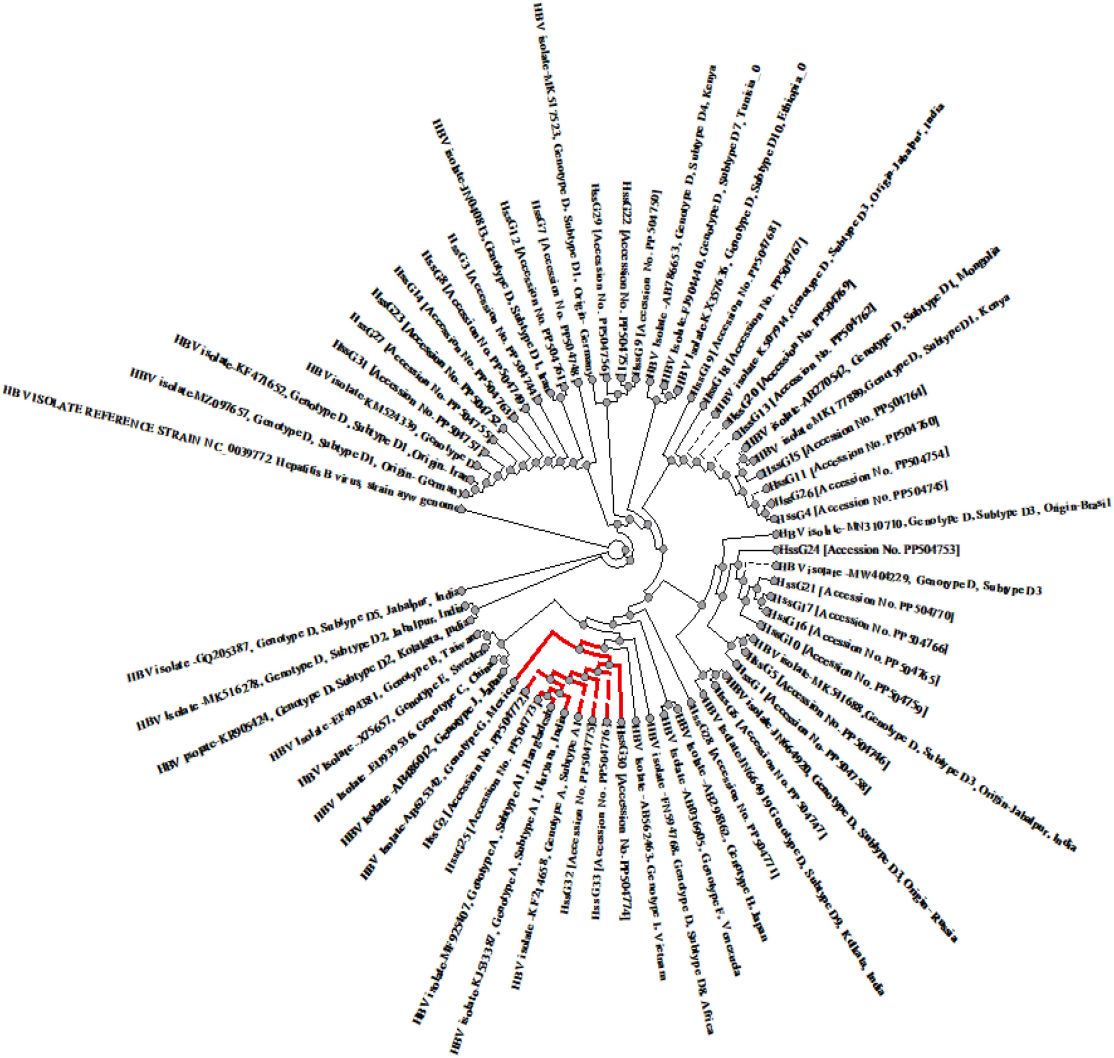
Phylogenetic analysis of isolates HSSG from Aligarh region were compared with reference strains from Genbank; highly homologous HBV reference sequences were chosen from GenBank for each genotype. Specifically, for genotype D, a total of 18 sequences were selected, comprising sub-genotypes D3 (5 sequences), D1(6 sequences), and D2, D4, D5, D6, D7, D8, D9, and D10 (1 sequence each). Additional reference sequences were obtained for genotypes A (2 sequences each), genotype B and C (1 sequence), genotypes E to J (1 sequences each). These sequences were aligned using ClustalW software. Genotype A is Depicted by red coloured branching. The phylogenetic tree was then constructed using the neighbour-joining method in UGENE software version 2.0. To assess the robustness of the tree, 1000 bootstraps were applied. Genetic distances were calculated using the Kimura 2-parameter model of nucleotide substitution, MEGA 4 package.

The analysis confirmed the presence of Genotype D in 35 samples (89.7%), with D1 and D3 subgenotypes equally represented. Genotype A (10.2%) was detected in 4 samples, all subgenotype A1. Genotype D sequences clustered with subgenotypes D2, D3, and D9 (from West Bengal), as well as D3 (from Russia, Jabalpur, and Brazil) (**Figure 6**). Some isolates also showed proximity to D1 strains reported from Iran, Germany, Kenya, and Mongolia, and to D4 from Kenya, indicating regional and international phylogenetic linkage.

## 4 Discussion

In this study, the HBV–HIV co-infection rate among ART-enrolled patients was 7.2%, which is comparable to reports from hospital-based studies in North India involving ART clinic populations, where prevalence rates of 5.3% (Gupta et al.), 9.9% (Jain et al.), and 11.3% (Saha et al.) have been documented, while a higher prevalence of 22.2% was reported by Sud et al., likely reflecting differences in study population characteristics, sampling strategies, and clinical settings (Chauhan et al., 2006; Gupta and Singh, 2006; Jindal et al., 2008; Jain et al., 2009; Saha et al., 2013; Chakravarty and Pal, 2015) (Gupta and Singh, 2006; Ruiz-Tachiquín et al., 2007; Jindal et al., 2008; Jain et al., 2009; Tamura et al., 2011; Chakravarty and Pal, 2015). Although this prevalence is lower than that reported from some high-endemic regions globally, it remains clinically and programmatically significant, given the well-established association of HBV–HIV co-infection with accelerated liver disease progression, increased risk of hepatocellular carcinoma, and greater complexity of antiviral management. In the era of widespread HAART, chronic viral hepatitis has emerged as a leading non-AIDS-defining cause of long-term morbidity and mortality, underscoring the importance of routine HBV screening and integrated management strategies within ART programs.

Consistent with previous studies, the mean CD4^+^ T-cell count was lower in HIV–HBV co-infected patients than in HIV mono-infected individuals. Reduced CD4^+^ T-cell counts reflect impaired immune surveillance, which may permit enhanced HBV replication and persistence, thereby contributing to adverse liver-related outcomes in HIV-HBV co-infected individuals.

The prevalence of HBeAg among HIV-HBV co-infected patients in this study was 17% as compared to HBV mono-infected patients in which it was 10%, however, this difference was not statistically significant. Koli et al. (Koli et al., 2016) reported a prevalence of 5.8% in HBV mono-infected individuals, whereas a study conducted in South India (Chandra et al., 2013) revealed a positivity rate of 21.4% among HIV-HBV co-infected patients. Given that HBeAg is a marker of active viral replication, the overall low HBeAg positivity in this cohort suggests that a substantial proportion of patients may have undergone HBeAg seroconversion, possibly influenced by immune status and antiretroviral therapy. Among the 39 HBV sequences analysed, genotype D predominated (89.7%), while genotype A accounted for 10.3%, a distribution broadly consistent with reports from North India (Thakur et al., 2002; Sami et al., 2013; Pacholi et al., 2022). Genotype A is common in northwestern Europe, while genotypes B and C dominate east Asia. However, genotype D is widespread (Thakur et al., 2002). Genotype D dominates northern, western, and southern India and is also found in Saudi Arabia, Iran, Afghanistan, Pakistan, and Turkey. The prevalence of genotype D is clinically significant, as this genotype correlates with reduced rates of spontaneous HBeAg seroconversion, extended immune-active phases, and an increased probability of progressive liver disease in comparison to genotype A. Genotype D also has more nucleotide variation in the surface gene, which could affect how HBsAg is expressed, how well it can escape the immune system, and how sensitive it is to laboratory tests. Conversely, genotype A, prevalent in northwestern Europe and certain regions of Africa, is associated with elevated HBeAg positivity rates, enhanced responses to interferon-based therapy, and expedited seroconversion, possibly indicating variations in viral replication dynamics and host–virus interactions. The significant prevalence of genotype D in this study has critical implications for disease progression, treatment response, and long-term clinical outcomes within this population. From an evolutionary and epidemiological perspective, the prevalence of genotype D in northern India likely signifies historical transmission dynamics and population migrations throughout the Middle East and South Asia, highlighting the necessity of region-specific molecular surveillance. Within genotype D, subgenotypes D1 and D3 were identified in this study. Subgenotype D1 is largely reported from the Mediterranean and Middle Eastern regions, whereas other genotype D subgenotypes (D2–D8) exhibit larger distribution across Africa, Eastern Europe, Australia, Indonesia, and India, reflecting a strong geographical pattern of HBV evolution. The finding of D1 and D3 in North India is consistent with regional molecular epidemiology and support historical and ongoing population movement impacting viral dissemination. All genotype A isolates belonged to subgenotype A1, which is the dominant A subgenotype reported from South Asia and parts of Africa (Ohba et al., 1995).

At the serological level, serotype ayw2 predominated, being present in nearly all genotype D strains, while serotype adw2 was exclusively associated with genotype A isolates. This distribution is consistent with well-established genotype–serotype linkages, whereby genotypes A, C, D, and F are typically associated with serotypes adw2, adr, ayw2/3, and adw4, respectively (Datta, 2008). In India, ayw and adw serotypes are most prevalent, with adr reported less frequently, particularly from southern regions (Thakur et al., 2002). The predominance of genotype D with serotype ayw2 and genotype A with serotype adw2 in the present study therefore aligns well with known regional patterns in North India.

HBV serotypes are determined by specific amino acid residues within the HBsAg, notably at positions 122, 127, 134/140, 159, 160, 177, and 178, which explains the close linkage between genotype and serotype. The consistent association of genotype D with serotype ayw2 and genotype A with serotype adw2 observed in this study reinforces the stability of this genotype–serotype relationship and supports the validity of the molecular and serological classification employed (Ohba et al., 1995).From an evolutionary perspective, the detection of genotype A (A1/adw2) in this population also mirrors patterns reported in Europe. Historical migration and long-standing population exchange between India and the United Kingdom over the past several centuries may have contributed to the introduction and persistence of genotype A strains in the Indian subcontinent. Together, these findings underscore the necessity of integrating genotype, subgenotype, and serotype data to better understand HBV transmission patterns, viral evolution, and region-specific clinical implications (Kao, 2002; Kramvis, 2014).

Mutations within the HBV surface (S) gene, particularly in the major hydrophilic region (MHR) and its ‘a’ determinant (amino acids 124–147), are of major clinical and public health significance. This region constitutes the primary target of neutralizing antibodies induced by both natural infection and hepatitis B vaccination. Amino acid substitutions within the ‘a’ determinant—most notably G145R, but also mutations at positions D144, P120, T126, and Q129—can alter the conformational epitope structure of HBsAg, resulting in reduced antibody binding, vaccine escape, and false-negative results in HBsAg-based diagnostic assays. The clinical implications of these mutations are substantial, as they may lead to occult HBV infection, ongoing viral transmission despite vaccination, and underestimation of true HBV prevalence due to diagnostic escape. From a public health perspective, the circulation of S gene escape variants underscores the need for continued molecular surveillance, periodic evaluation of diagnostic assays, and potential updates to vaccine design to ensure sustained effectiveness of HBV control strategies, particularly in high-burden settings (Carman et al., 1990; Banerjee et al., 2006; Salpini et al., 2015).In this study, nucleotide substitutions within the “a” determinant were observed in 7 (36.8%) HBV-HIV co-infected patients. No such mutations were identified in HBV mono-infected individuals. Of these, two were nonsynonymous (amino acid-altering), while five were synonymous. In addition to impairing antibody-mediated neutralization, these mutations pose challenges for HBV control by potentially undermining the effectiveness of passive immunisation strategies and reducing the sensitivity of routine HBsAg-based diagnostic assays. Their emergence in HIV-HBV co-infected populations underscores the importance of sustained molecular surveillance in this group to accurately monitor viral evolution and ensure the continued effectiveness of preventive and diagnostic interventions (Pondé RA de and Amorim G de, 2024). Additional mutations in pre-S and S regions were linked to higher ALT/AST levels and elevated APRI and FIB-4 scores, suggesting a potential association with liver disease progression. Specific mutations—preS1-A482C, S-A644C, and S-G684A—were observed in patients with high fibrosis scores, though no statistically significant association could be confirmed due to limited sample size. Other mutations such as S-C198A, S-G195T, preS-C162T, and preS-A163G were significantly associated with deranged liver function tests and were more common in genotype A cases, warranting further investigation in larger cohorts. These mutations may impair antibody recognition, leading to vaccine escape, failure of passive immunisation, and false-negative serological results and suggest the need for continued molecular surveillance in HIV-HBV co-infected populations. Interestingly, pre-S amino acid insertions identified in this study did not correlate with liver disease severity, unlike previous reports (Chen, 2018). HBeAg positivity was associated with the S-G189A mutant, suggesting a possible role in viral replication or immune modulation.

While several of the detected S and pre-S mutations have established roles in immune escape and liver disease in other settings, the associations observed in this study should be interpreted cautiously. They highlight potential mutation hotspots in HIV–HBV co-infection that warrant further investigation using larger, longitudinal cohorts and functional assays.

The limitations of this study include the small sample size, small S-gene coverage, lack of longitudinal follow-up, and the use of a genotyping assay designed to detect HBV genotypes A–H, which may not identify the rare genotypes I and J. However, given the absence of reports of these genotypes from northern India, their omission is unlikely to materially affect the interpretation of the findings. The genotype distribution, serotype frequencies, and prevalence of surface gene mutations reported here may not be fully representative of the entire study population, particularly in patients with low-level viremia. However, the observed predominance of genotype D and the higher frequency of ‘a’ determinant mutations in HBV–HIV co-infected patients remain clinically relevant, as individuals with higher viral loads are at increased risk of transmission, disease progression, and diagnostic or vaccine escape. Furthermore, the use of consecutive, non-probability sampling may limit the generalizability of the findings beyond the hospital setting, as the study population may not fully represent the broader community of HBV-infected individuals.

## 5 Conclusion

Shared pathways of transmission and synergistic effects of both viruses have an impact on morbidity and mortality of HIV. Screening of HBV should be included in the investigations to be done pre-HAART. This study highlights genotype D predominance and a higher frequency of immune escape mutations in HBV-HIV co-infected patients. The exclusive presence of serotype ayw2 across all genotype D isolates and the detection of S gene mutations suggest the need for continued molecular surveillance in HIV-HBV co-infected populations.

## Data Availability

The datasets presented in this study can be found in online repositories. The names of the repository/repositories and accession number(s) can be found in the article/**Supplementary Material**.
