# Supplementary material for: HBV genotype distribution and S gene mutations in HIV-HBV co-infected patients: insights from North India
Source: Front Cell Infect Microbiol. 2026 Feb 9;15:1731472. doi: 10.3389/fcimb.2025.1731472 (PMC12926437; doi:10.3389/fcimb.2025.1731472)
Supplement: Supplementary file 1 [file Table1.docx]

**Supplementary Table 1: Comparison of Genetic Variability Measures between Genotype A and Genotype D.**

| Variability measures | Genotype-D | Genotype-A |
| --- | --- | --- |
| Average ps | 0.724069 | 0.718154 |
| Variance (ps) | 0.000644 | 0.000556 |
| Std Deviation(ps) | 0.025385 | 0.023584 |
| Average ds | Nan | Nan |
| Variance ds | Nan | Nan |
| Std Deviation(ds) | Nan | Nan |
| Average pn | 0.747811 | 0.713992 |
| Variance(pn) | 0.000187 | 0.000154 |
| Std Deviation(pn) | 0.013684 | 0.012412 |
| Average dn | Nan | 2.299984 |
| Variance(dn) | Nan | 0.153858 |
| Std Deviation(dn) | Nan | 0.392247 |
