# Supplementary material for: HBV genotype distribution and S gene mutations in HIV-HBV co-infected patients: insights from North India
Source: Front Cell Infect Microbiol. 2026 Feb 9;15:1731472. doi: 10.3389/fcimb.2025.1731472 (PMC12926437; doi:10.3389/fcimb.2025.1731472)
Supplement: Supplementary file 2 [file Table2.docx]

|  | HIV-HBV Co-infected (Surface Gene Detected) (n=19) | HIV-HBV Co-infected (Surface Gene Not-Detected) (n=81) | HBV mono-infected Surface Gene Detected(n=20) | HBV mono-infected Surface Gene Not-Detected(n=30) |
| --- | --- | --- | --- | --- |
| Age (Mean) | 36.06±10.03 | 36.59±10.04 | 34.92±14.84 | 36.09±15.22 |
| Gender | M:F:T= 13:5:1 | M:F=19:8 | M:F=12:9 | M:F= 19:10 |
| ALT Mean (95%CI) | 62.22 IU/L | 62.13 IU/L | 127.11 IU/L | 131.35 IU/L |
| AST Mean (95%CI) | 56.26 IU/L | 55.97 IU/L | 97.51 IU/L | 100.26 IU/L |
| Alk. P Mean (95%CI) | 166.97 IU/L | 167.54 IU/L | 179.58 IU/L | 180.3 IU/L |
| APRI Score | 0.56 | 0.57 | 1.31 | 1.34 |
| FIB-4 | 1.63 | 1.64 | 2.10 | 2.15 |
| HBeAg positivity |  |  |  |  |
| Positive | 12 (63.1%) | 5 (6.1%) | 4 (20%) | 1 (33.3%) |
| Negative | 7 (36.8%) | 76 (93.8%) | 16 (80%) | 29 (96.6%) |
| Mean HBV Viral Load (IU/mL) | 2.84 × 10⁸ IU/ml | <20 IU/ml | 3.20 ×10⁸ IU/ml | <20 IU/ml |
| ART Duration |  |  |  |  |
| Treatment Naive | 2 (10.5%) | 7 (8.6%) | - | - |
| < 6 Months | 4 (21.05%) | 6 (7.4%) | - | - |
| 6-12 Months | 1 (5.2%) | 10 (12.3%) | - | - |
| > 12 months | 12 (63.2%) | 58 (71.6%) | -- | - |

M=Male;F=Female;T=Trangender

**Supplementary Table 2: Baseline clinical and virological characteristics of sequenced versus non-sequenced HBV samples in HIV–HBV co-infected and HBV mono-infected patients.**
