# Supplementary material for: HBV genotype distribution and S gene mutations in HIV-HBV co-infected patients: insights from North India
Source: Front Cell Infect Microbiol. 2026 Feb 9;15:1731472. doi: 10.3389/fcimb.2025.1731472 (PMC12926437; doi:10.3389/fcimb.2025.1731472)
Supplement: Supplementary file 3 [file Supplementaryfile1.docx]

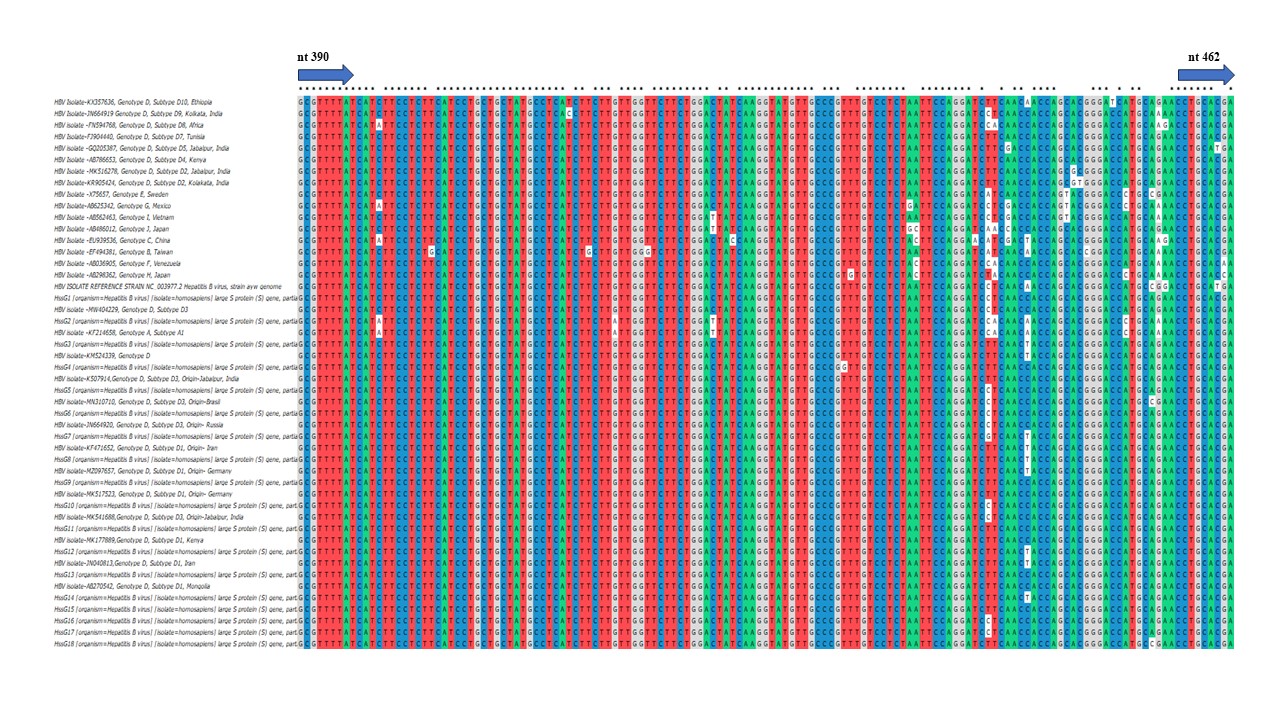


**Supplementary Figure 1:** The multiple alignment of the inner region (480bp) *(nt 390 to nt 462)* of the surface gene of HBV in study samples and genotype specific wild-type references using UGENE software. The star symbol represents areas of nucleotide similarity while its absence signifies nucleotide variation
